# Whole-Food Dietary Interventions in Crohn’s Disease: A Literature Review

**DOI:** 10.7759/cureus.103488

**Published:** 2026-02-12

**Authors:** Abdulrahman Al-Majmuei, Farah Fakhri, Lyaba Imran

**Affiliations:** 1 Medicine, Royal College of Surgeons in Ireland, Manama, BHR; 2 Internal Medicine, Royal College of Surgeons in Ireland, Manama, BHR

**Keywords:** crohn’s disease, crohn’s disease exclusion diet, dietary therapy, enteral nutrition, inflammatory bowel disease, mediterranean diet, nutrition therapy, whole-food diets

## Abstract

This narrative review examines advances in diet-based therapy for Crohn’s disease with a focus on emerging whole-food interventions that have gained attention in the past five years. The aim is to evaluate the current evidence for the Crohn’s disease exclusion diet, specific carbohydrate diet, Mediterranean diet, and newer approaches such as CD-TREAT (Crohn’s disease treatment with eating diet), low-FODMAP (fermentable oligosaccharides, disaccharides, monosaccharides and polyols) strategies, and plant-based regimens. Drawing on recent randomised controlled trials and mechanistic studies, this review explores how these diets influence clinical outcomes, gut microbiota composition, and mucosal integrity, while identifying limitations, controversies, and gaps in knowledge. In addition to summarising the latest findings, we offer a critical perspective on the potential for personalised nutrition, the integration of diet with pharmacologic therapy, and the need for long-term maintenance studies. The scope is limited to mild-to-moderate Crohn’s disease, reflecting the clinical scenarios where dietary interventions are most often applied. This review emphasises the potential for diet to function as a structured therapeutic intervention aligned with patient preferences, provided it is implemented within multidisciplinary care models to ensure efficacy, safety, and sustainability.

## Introduction and background

Crohn’s disease is a chronic, relapsing inflammatory bowel disease (IBD) characterised by transmural intestinal inflammation arising from a complex interaction between genetic susceptibility, immune dysregulation, the gut microbiome, and environmental exposures, of which diet is a central component [1,2]. Its global burden continues to rise, particularly in newly industrialised regions, placing increasing strain on healthcare systems and long-term patient management [3,4]. Beyond its biological complexity, Crohn’s disease imposes a substantial daily burden on patients, often affecting nutrition, social functioning, and quality of life. Consequently, many patients actively seek nutritional strategies that provide a sense of disease control alongside conventional medical therapy. Pharmacological therapies such as corticosteroids, immunomodulators, and biologic agents remain the cornerstone of treatment. However, many patients experience incomplete response, loss of efficacy over time, or adverse effects, and up to one-third fail to achieve primary response to biologic therapy, with secondary loss of response occurring in a substantial proportion [5]. These therapeutic limitations, together with patient concerns regarding long-term immunosuppression and overall treatment burden, have intensified interest in adjunctive non-pharmacological approaches, particularly diet-based interventions [5].

Diet is uniquely positioned to influence Crohn’s disease activity through its direct and indirect effects on intestinal physiology. It shapes the gut microbiome, alters microbial metabolic outputs, modulates epithelial barrier integrity, and influences mucosal immune responses [6]. Western-style dietary patterns, characterised by high intakes of saturated fat, refined sugars, and ultra-processed foods, have been associated with dysbiosis, increased intestinal permeability, and pro-inflammatory immune activation [7,8]. In contrast, nutritional interventions that limit these exposures or promote microbiome-supportive nutrients have demonstrated the capacity to modify inflammatory pathways relevant to Crohn’s disease [9]. This biological plausibility provides a strong rationale for investigating diet not merely as symptom management, but as a potential disease-modifying intervention.

Historically, exclusive enteral nutrition (EEN) provided the clearest proof of concept that dietary manipulation alone can induce remission in Crohn’s disease, particularly in paediatric populations [10]. However, the poor palatability and social burden of an all-formula regimen have limited its widespread adoption in adults and long-term use [11]. In response, recent years have seen the development of whole-food dietary strategies designed to replicate the anti-inflammatory effects of EEN while improving acceptability and sustainability. These approaches include structured exclusion diets, pattern-based dietary models, and food-based analogues of enteral nutrition, each targeting overlapping but distinct nutritional and microbial pathways [12,13].

Despite growing interest, the current literature on diet-based therapy in Crohn’s disease remains fragmented. Many existing reviews focus on individual nutritional interventions in isolation, combine paediatric and adult data without clear clinical stratification, or emphasise symptomatic outcomes without integrating mechanistic insights [14,15]. Furthermore, few reviews clearly distinguish between strategies used for induction of remission and those intended for long-term maintenance, or critically address the heterogeneity of patient response and the limitations of existing trials [16,17]. As a result, clinicians are often left without clear guidance on how to position diet-based therapy within contemporary Crohn’s disease management [18].

This narrative review aims to address these gaps by synthesising recent evidence on nutritional therapies for Crohn’s disease published over the past five years, with an emphasis on whole-food interventions. We integrate clinical trial data with mechanistic findings related to the gut microbiome, intestinal barrier function, and immune modulation, while critically evaluating the strengths, limitations, and clinical applicability of each dietary approach. Particular attention is given to comparative effectiveness, patient adherence, and the role of diet as an adjunct rather than a replacement for pharmacological therapy.

The scope of this review is limited to mild-to-moderate Crohn’s disease because nutritional interventions are most feasible and safest within this clinical context. Patients with severe, stricturing, or penetrating disease typically require prompt pharmacological escalation or surgical management, whereas dietary strategies may offer greater therapeutic flexibility and patient acceptability earlier in the disease course [18]. This review first examines EEN as a therapeutic benchmark, followed by evaluation of emerging whole-food dietary interventions, their underlying biological mechanisms, current limitations, and future directions for clinical integration. By clarifying where these strategies show promise, where evidence remains insufficient, and how such interventions may be integrated into personalised care, the aim is to provide clinicians and researchers with a balanced, evidence-informed framework for the evolving role of diet in Crohn’s disease management. 

Methods 

Literature Search Strategy

A structured and comprehensive literature search was performed to identify relevant studies evaluating dietary interventions in Crohn’s disease. Electronic databases, including PubMed, Scopus, and Web of Science, were systematically searched to capture a broad representation of biomedical and clinical nutrition research. The search was restricted to articles published in English between January 2019 and December 2025 in order to emphasise contemporary developments in whole-food dietary therapy. This timeframe was selected because the majority of structured dietary interventions, particularly exclusion-based and whole-food analogue strategies, have been developed and evaluated within the past five years. However, landmark clinical trials, foundational mechanistic studies, and historically influential publications predating this period were also reviewed, where necessary, to provide biological and clinical context for emerging therapeutic models.

The search strategy was developed using combinations of Medical Subject Headings and free-text keywords. Search terms included variations and combinations of “Crohn’s disease,” “inflammatory bowel disease,” “diet therapy,” “dietary intervention,” “nutrition therapy,” “exclusive enteral nutrition,” “Crohn’s Disease Exclusion Diet,” “specific carbohydrate diet,” “Mediterranean diet,” “CD-TREAT,” “low fermentable oligosaccharides disaccharides monosaccharides and polyols,” “microbiome,” “intestinal permeability,” “epithelial barrier function,” and “faecal calprotectin.” Boolean operators and database-specific indexing filters were applied where appropriate to refine search results and improve relevance. In addition to electronic database searches, reference lists of eligible articles, relevant systematic reviews, international clinical guidelines, and pivotal randomised controlled trials were manually screened to identify additional studies that may not have been captured through database searching alone.

Study Selection and Eligibility Criteria

Studies were considered eligible for inclusion if they evaluated dietary interventions intended to influence Crohn’s disease activity and reported clinically relevant outcomes. Eligible studies included randomised controlled trials, prospective cohort studies, controlled observational studies, systematic reviews, and mechanistic investigations examining the biological effects of dietary therapy. Both adult and paediatric populations were included in order to provide a comprehensive overview of therapeutic trends and to allow comparison of evidence across age groups. Studies were required to report at least one of the following outcome domains: clinical remission or disease activity indices, inflammatory biomarkers including C-reactive protein (CRP) or faecal calprotectin, endoscopic or mucosal healing outcomes, microbiome composition or metabolic changes, or measures of intestinal barrier integrity.

Studies were excluded if they focused exclusively on ulcerative colitis or other non-Crohn’s IBDs, investigated functional gastrointestinal disorders without inflammatory disease activity, examined parenteral nutrition without an oral or enteral dietary intervention component, or evaluated complementary or alternative therapies that lacked a clearly defined nutritional intervention. Case reports, editorials, opinion pieces, and studies with insufficient methodological detail were also excluded to maintain scientific robustness. Particular emphasis was placed on studies involving patients with mild-to-moderate Crohn’s disease, as dietary therapy is most commonly implemented and most clinically feasible within this disease severity spectrum. Evidence involving severe, stricturing, or penetrating disease phenotypes was reviewed but interpreted cautiously due to differences in therapeutic requirements and safety considerations.

Study Selection Process

Following completion of database searches, titles and abstracts of identified studies were screened to determine preliminary relevance to the research question. Study identification, screening, and inclusion decisions were performed by two reviewers (AA and FF), with uncertainties resolved through discussion with the third reviewer (LI). Studies meeting initial eligibility criteria underwent full-text review to confirm inclusion suitability. When multiple publications reported outcomes from overlapping study populations, priority was given to the most comprehensive, recent, or methodologically rigorous dataset to minimise duplication of evidence. 

Data Extraction and Evidence Evaluation

Relevant data were extracted from eligible studies focusing on study design, patient population characteristics, dietary intervention protocols, duration of intervention, clinical and biochemical outcomes, mechanistic findings, adherence measures, and reported limitations. Particular attention was given to the methodological quality of included studies, including sample size, follow-up duration, use of objective inflammatory endpoints, and reproducibility of dietary protocols. Randomised controlled trials and prospective studies were prioritised in evidence synthesis, while observational studies and pilot investigations were included primarily to support mechanistic interpretation and emerging clinical trends.

Data Synthesis and Analytical Framework

Due to significant heterogeneity across dietary interventions, including variation in dietary composition, implementation protocols, comparator groups, and outcome measurement tools, formal quantitative meta-analysis was not considered methodologically appropriate. Instead, findings were synthesised narratively using thematic analysis to identify shared mechanistic pathways, comparative clinical effectiveness, and practical considerations for implementation. Dietary interventions were grouped according to therapeutic design and clinical intent, including structured exclusion-based diets, whole-food analogue diets replicating enteral nutrition, and symptom-directed dietary strategies. Mechanistic findings were integrated across studies to identify convergent biological pathways linking dietary modification with modulation of intestinal inflammation.

Risk of Bias and Methodological Limitations

Formal risk-of-bias scoring tools were not applied due to the narrative review design and the broad heterogeneity of included study types. However, methodological quality was considered during evidence interpretation. Greater weight was assigned to randomised controlled trials and prospective cohort studies with clearly defined intervention protocols and objective inflammatory endpoints. Observational studies, pilot trials, and mechanistic investigations were interpreted cautiously and primarily used to support hypothesis generation and biological plausibility. Limitations, including small sample sizes, short follow-up durations, variability in adherence reporting, and inconsistency in outcome measurement, were critically evaluated and discussed within the manuscript to provide a balanced interpretation of the available evidence.

## Review

EEN: Benchmark and limitations

EEN involves the provision of a nutritionally complete liquid formula as the sole source of caloric intake for a defined period, typically six to eight weeks [19]. It remains one of the most effective non-pharmacological interventions for inducing remission in Crohn’s disease, particularly in paediatric populations, where remission rates comparable to corticosteroids and higher rates of mucosal healing have been consistently reported [20,21]. The success of EEN established a critical proof of concept: dietary manipulation alone can suppress intestinal inflammation in Crohn’s disease [20,21].

The therapeutic effects of EEN are thought to arise from several converging mechanisms. By completely excluding whole foods, EEN markedly reduces exposure to dietary antigens, food additives, and complex substrates that may fuel dysbiosis [17]. This dietary simplification leads to rapid and profound alterations in the gut microbiome, including a reduction in microbial diversity and overall bacterial load [17]. Although reduced diversity is often considered unfavourable in health, short-term microbial suppression during active inflammation may decrease antigenic stimulation of the mucosal immune system [22,23]. In parallel, EEN has been shown to reduce intestinal permeability, modulate bile acid profiles, and lower inflammatory biomarkers such as CRP and faecal calprotectin, supporting its role as a potent induction therapy [22,23].

Despite its efficacy, EEN has significant practical limitations that restrict its broader use. Adherence is a major barrier, particularly in adolescents and adults, due to poor palatability, social isolation associated with exclusive liquid intake, and difficulty maintaining normal daily routines [21]. Real-world studies consistently demonstrate lower compliance outside controlled trial settings, and relapse is common once a normal diet is reintroduced [24]. Furthermore, EEN is primarily used as a short-term induction strategy and is rarely sustained as long-term maintenance therapy due to its restrictive nature [25].

These limitations have important implications for the evolution of diet-based therapies in Crohn’s disease. While EEN remains a valuable first-line option in paediatric practice, its constraints highlight the need for more acceptable dietary approaches that retain anti-inflammatory efficacy while allowing the inclusion of whole foods. This recognition directly informed the development of newer dietary strategies, including structured exclusion diets and food-based analogues of enteral nutrition, which aim to replicate the beneficial mechanistic effects of EEN without requiring complete food elimination [26].

**Figure 1:**
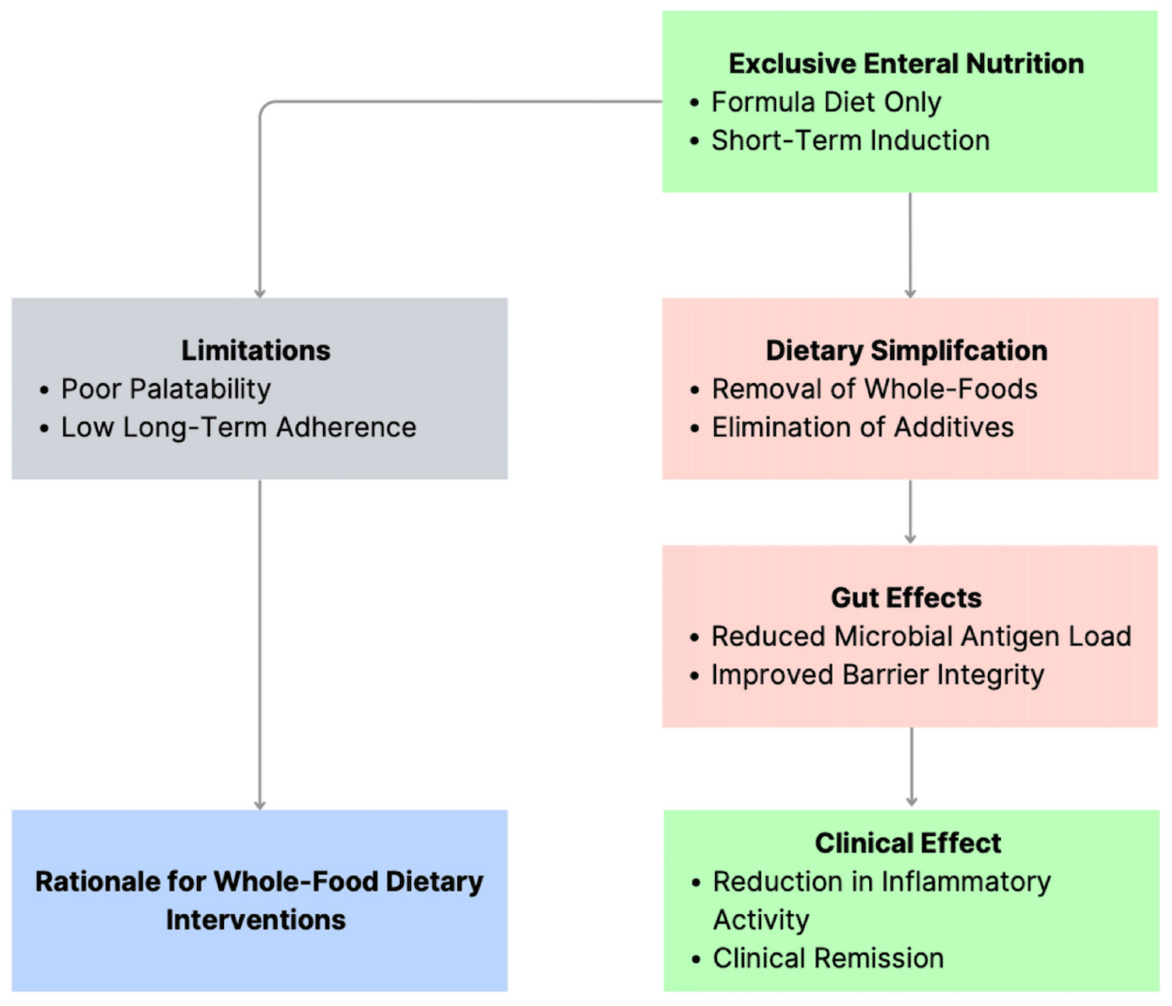
Exclusive enteral nutrition as a proof-of-concept for diet-based therapy in Crohn’s disease. This schematic was created by the authors using Microsoft PowerPoint.

Accordingly, in the context of this review, EEN is best viewed not as a competing dietary option but as a benchmark against which emerging whole-food interventions can be evaluated. Its success demonstrates that diet can exert disease-modifying effects in Crohn’s disease, while its limitations underscore the importance of sustainability, adherence, and long-term applicability when translating dietary therapies into routine clinical practice. The mechanistic rationale of EEN is illustrated in Figure 1.

Emerging whole-food dietary interventions in Crohn’s disease

Emerging whole-food dietary interventions in Crohn’s disease share a unifying principle: selective exclusion of dietary components thought to exacerbate intestinal inflammation, coupled with the inclusion of nutritionally adequate, minimally processed foods [17]. Rather than relying on complete food elimination, these approaches seek to modify the intestinal environment through targeted dietary manipulation, positioning diet as a therapeutic tool rather than solely a means of symptom control [17].

Whole-food dietary interventions differ in their degree of restriction, mechanistic emphasis, and clinical intent. Some are designed primarily for induction of remission, closely mirroring the effects of EEN, while others are better suited for maintenance or symptom modulation [24]. Despite these differences, common mechanistic themes emerge across dietary strategies, including reduction of exposure to ultra-processed foods and food additives, modulation of gut microbial composition and metabolic activity, and preservation of intestinal barrier integrity [24]. These shared pathways provide a biological rationale for evaluating such diets within a unified framework rather than as isolated interventions.

A key distinction between whole-food dietary strategies and traditional exclusion diets is their emphasis on dietary pattern rather than single nutrient avoidance [17,18]. Instead of targeting individual macronutrients, many of these interventions focus on removing components characteristic of Western-style diets, such as refined sugars, emulsifiers, and excessive saturated fats, while prioritising whole foods that support microbial diversity and mucosal health [17,18]. This shift reflects growing recognition that overall dietary quality and food processing may be more relevant to Crohn’s disease activity than isolated dietary constituents.

Clinical evidence supporting whole-food dietary interventions has expanded in recent years, particularly in populations with mild-to-moderate Crohn’s disease. Randomised controlled trials and prospective cohort studies demonstrate that structured dietary approaches can induce clinical remission in a subset of patients, with accompanying reductions in inflammatory biomarkers [14,18]. However, responses are heterogeneous, and remission rates vary across interventions, highlighting that diet alone is unlikely to be universally effective. Importantly, many studies report improved tolerability and adherence compared with EEN, suggesting that sustainability may represent a key advantage of whole-food approaches even when efficacy is comparable [27].

Whole-food dietary interventions also raise important considerations regarding clinical positioning. While some diets show promise as induction strategies in carefully selected patients, others appear better suited as adjunctive therapies alongside pharmacological treatment or as maintenance strategies following remission [28]. The majority of available data support their use in mild-to-moderate disease, with limited evidence in severe or complicated Crohn’s disease. Accordingly, these diets should be viewed as complementary to, rather than replacements for, established medical therapies, particularly in patients with aggressive disease phenotypes.

Within this evolving landscape, the Crohn’s disease exclusion diet (CDED) has emerged as the most extensively studied whole-food dietary intervention, with evidence supporting its use for both induction and maintenance of remission [14]. Its structured design, mechanistic rationale, and comparative trial data distinguish it from less standardised dietary approaches. The following section examines CDED in detail, highlighting its clinical evidence, proposed mechanisms of action, and practical considerations for implementation.

Crohn’s Disease Exclusion Diet

CDED has emerged as the most rigorously studied whole-food dietary intervention for Crohn’s disease and represents a pragmatic evolution of diet-based therapy beyond EEN. Developed to replicate the anti-inflammatory effects of enteral feeding while allowing the inclusion of whole foods, CDED is a structured, phased dietary programme that systematically excludes dietary components implicated in intestinal inflammation and barrier disruption [14,29].

CDED is typically implemented over an initial induction phase followed by a transition and maintenance phase [14,29]. During induction, patients consume a restricted selection of whole foods alongside partial enteral nutrition (PEN), with the aim of maximising anti-inflammatory efficacy while maintaining nutritional adequacy [14,29]. Subsequent phases gradually reintroduce additional foods in a controlled manner, supporting long-term adherence and sustainability [14,29]. 

Clinical evidence of CDED: The strongest evidence for CDED comes from a multicentre randomised controlled trial in paediatric patients with mild-to-moderate Crohn’s disease, in which CDED combined with PEN was compared directly with EEN [14]. At six weeks, rates of clinical remission were comparable between groups; however, by 12 weeks, sustained remission was significantly higher in the CDED group [14]. Importantly, adherence was substantially better with CDED, highlighting its improved tolerability relative to exclusive liquid feeding [14]. Reductions in inflammatory biomarkers, including CRP and faecal calprotectin, accompanied clinical response, supporting a genuine anti-inflammatory effect rather than symptomatic improvement alone [14].

Subsequent studies have extended these findings to adult populations. An open-label trial in biologic-naïve adults with mild-to-moderate Crohn’s disease demonstrated that a substantial proportion of participants achieved clinical remission by six weeks on CDED, with many maintaining remission through extended follow-up when the diet was continued [27,29]. Although sample sizes were modest and endoscopic endpoints were limited, these studies provide important proof-of-concept that the benefits of CDED are not restricted to paediatric disease.

Across available trials, several consistent themes emerge. First, CDED appears effective as an induction strategy in selected patients, with remission rates comparable to more restrictive approaches [14]. Second, its structured reintroduction phase supports maintenance of response, addressing a key limitation of EEN [14]. Third, successful implementation typically requires dietitian support and patient engagement, underscoring that CDED is a therapeutic intervention rather than a simple dietary preference [27,30].

Mechanistic rationale of CDED: The proposed mechanisms underlying CDED centre on the selective exclusion of dietary elements associated with Westernised eating patterns, particularly ultra-processed foods, emulsifiers, maltodextrin, and excess saturated fat [14]. Experimental and epidemiological data suggest that these components may disrupt the intestinal mucus layer, increase epithelial permeability, and promote dysbiosis characterised by expansion of pro-inflammatory bacterial taxa [31,32].

Notably, the mechanistic rationale of CDED aligns closely with the conceptual lessons derived from EEN. Both approaches achieve dietary simplification and exclusion of potentially pro-inflammatory components; however, CDED does so in a manner that is compatible with long-term use [14,33]. This balance between efficacy and sustainability represents one of the central strengths of the diet and explains its growing clinical interest.

Taken together, available evidence positions CDED as the most robustly supported whole-food dietary intervention for Crohn’s disease to date. While it is unlikely to replace pharmacological therapy in patients with severe or refractory disease, it offers a viable option for induction and maintenance of remission in selected individuals with mild-to-moderate disease, particularly when implemented within a multidisciplinary care framework.

Comparator Whole-Food Dietary Patterns

Several whole-food dietary patterns have been evaluated in Crohn’s disease and are frequently cited as alternatives or comparators to more structured exclusion-based approaches. While these diets share conceptual overlap with CDED in their emphasis on unprocessed foods and dietary quality, their clinical evidence base, mechanistic specificity, and therapeutic positioning differ substantially. Rather than serving as direct substitutes for CDED, these dietary patterns are best understood as contextual comparators that inform the broader role of diet in disease management.

Specific carbohydrate diet and Mediterranean diet: The specific carbohydrate diet (SCD) and Mediterranean diet represent two widely studied structured dietary patterns that differ substantially in their degree of restriction, mechanistic targeting, and clinical applicability. Both diets aim to improve intestinal health through modification of dietary composition and reduction of ultra-processed food exposure, yet they approach this objective through distinct nutritional frameworks [18].

SCD is based on the exclusion of complex carbohydrates, grains, and most dairy products. The theoretical rationale underlying this approach proposes that poorly absorbed carbohydrates promote dysbiosis, luminal fermentation, and downstream mucosal inflammation [34]. Early observational studies and patient-reported outcome data suggested symptomatic improvement in some individuals; however, these studies frequently lacked objective inflammatory endpoints and were limited by small sample sizes and heterogeneous methodology [34]. As a result, the overall strength of evidence supporting SCD as a disease-modifying intervention has remained uncertain.

The Mediterranean diet, in contrast, represents a broadly anti-inflammatory dietary pattern characterised by high consumption of fruits, vegetables, whole grains, legumes, olive oil, and fish, alongside reduced intake of red meat and ultra-processed foods [35]. Rather than selectively excluding specific macronutrients, the Mediterranean diet emphasises overall dietary quality and balance. This pattern has been extensively studied in cardiometabolic disease and is associated with favourable effects on microbial diversity and systemic inflammation [35]. Its relatively low restrictiveness and cultural adaptability contribute to strong long-term adherence in clinical settings [35].

Direct comparative evidence between these dietary approaches is provided by the DINE-CD randomised controlled trial, which evaluated adults with mild-to-moderate Crohn’s disease assigned to either SCD or the Mediterranean diet [18]. At both six and 12 weeks, rates of symptomatic remission were similar between the two groups, with no statistically significant differences observed in inflammatory biomarkers or quality-of-life measures [18]. Importantly, adherence rates were significantly higher among participants assigned to the Mediterranean diet, highlighting a key limitation of SCD related to its restrictive nature and challenges in long-term sustainability [18]. Consequently, the Mediterranean diet may serve as a pragmatic baseline dietary pattern for patients with Crohn’s disease, particularly as a maintenance strategy or adjunct to pharmacological therapy, while SCD may be considered in carefully selected patients who demonstrate individual symptomatic responsiveness.

Whole-food analogues of enteral nutrition: CD-TREAT: The Crohn’s disease treatment with eating diet (CD-TREAT) represents a novel dietary strategy developed as a whole-food analogue of EEN. This approach was specifically designed to replicate the nutritional composition and biological effects of enteral formula therapy while allowing patients to consume ordinary foods [36]. The underlying rationale of CD-TREAT is to reproduce the anti-inflammatory and microbiome-modulating properties of EEN by carefully matching macronutrient composition and excluding dietary components believed to contribute to mucosal inflammation [36].

Early clinical investigations, primarily conducted in paediatric populations, have demonstrated encouraging clinical and biochemical responses associated with CD-TREAT implementation. These studies have reported reductions in inflammatory biomarkers and disease activity indices, alongside favourable tolerability compared with exclusive liquid feeding [36]. Mechanistic analyses suggest that CD-TREAT produces metabolic and microbial alterations comparable to those observed during EEN, supporting its conceptual role as a functional dietary analogue [36].

Despite these promising findings, the current evidence base remains limited. Most available studies are small pilot trials with relatively short follow-up durations and limited endoscopic outcome data. Additionally, adequately powered randomised controlled trials directly comparing CD-TREAT with established dietary or pharmacological therapies remain scarce. Consequently, although CD-TREAT represents an innovative and biologically plausible intervention, further research is required to clarify its long-term efficacy, durability of remission, and optimal positioning within clinical management pathways.

Symptom-directed dietary strategies: Several dietary interventions have been evaluated in Crohn’s disease primarily for symptom management rather than disease modification. Among these, low fermentable oligosaccharides, disaccharides, monosaccharides, and polyols (FODMAP) dietary restriction and fibre-modulated interventions have received particular attention.

Low-FODMAP diets are designed to reduce poorly absorbed fermentable carbohydrates that contribute to luminal gas production, bloating, abdominal pain, and diarrhoea [37]. Multiple clinical studies have demonstrated that these diets can significantly improve functional gastrointestinal symptoms, particularly in patients with Crohn’s disease who have overlapping irritable bowel-type features [37,38]. However, available evidence does not consistently demonstrate reductions in inflammatory biomarkers or objective disease activity indices. As a result, low-FODMAP dietary restriction is generally considered a symptom-focused intervention rather than a disease-modifying therapy [38].

Fibre-focused dietary interventions have also been investigated, with variable findings depending on disease phenotype, inflammatory burden, and individual patient tolerance. Some studies suggest that specific fibre types may promote short-chain fatty acid production and support mucosal health, whereas others report limited or inconsistent clinical benefit [39,40]. The heterogeneity of fibre sources and intervention protocols, combined with limited high-quality trial data, currently restricts definitive conclusions regarding routine therapeutic use.

Collectively, these dietary approaches have contributed valuable insights into diet-microbiome-host interactions in Crohn’s disease but remain constrained by limited mechanistic coherence, inconsistent inflammatory outcomes, and narrow clinical applicability. In contrast, CDED is distinguished by its structured, mechanism-driven design, with supportive clinical trial evidence demonstrating efficacy in both induction and maintenance settings. This combination of biological plausibility, clinical effectiveness, and practical applicability positions CDED as the most robust whole-food dietary intervention currently available.

Key clinical trial data supporting whole-food dietary strategies are summarised in Table 1, with underlying biological mechanisms synthesised in Table 2. 

**Table 1:** Key Clinical Trials of Whole-Food Dietary Interventions in Crohn’s Disease CDED, Crohn’s disease exclusion diet; CD-TREAT, Crohn’s disease treatment-with-eating diet; EEN, exclusive enteral nutrition. Note: The symbol ± indicates that the Crohn’s disease exclusion diet was implemented with or without partial enteral nutrition, depending on the study protocol.

Diet Intervention	Study Population	Key Clinical Outcomes	Reference
Crohn’s disease exclusion diet (CDED) ± partial enteral nutrition	Paediatric and adult mild-to-moderate Crohn’s disease	Induction and maintenance of remission; improved tolerability compared with EEN	[9]
Specific carbohydrate diet versus Mediterranean diet	Adults with mild-to-moderate Crohn’s disease	No significant difference in remission or inflammatory markers; Mediterranean diet better tolerated	[13]
Whole-food diet mimicking EEN (CD-TREAT)	Children with active Crohn’s disease (pilot study)	Clinical improvement with reductions in inflammatory markers in small pilot cohorts	[22]

**Table 2:** Proposed Effects of Whole-Food Dietary Interventions in Crohn’s Disease CDED, Crohn’s disease exclusion diet; SCD, specific carbohydrate diet; CD-TREAT, Crohn’s disease treatment-with-eating diet; FODMAP, fermentable oligosaccharides, disaccharides, monosaccharides and polyols; EEN, exclusive enteral nutrition.

Diet	Core Dietary Features	Reported Biological Themes	Clinical Context and Interpretation	References
Crohn’s disease exclusion diet (CDED)	Excludes ultra-processed foods with phased whole-food reintroduction	Gut microbial shifts and barrier-related improvements reported	Associated with sustained remission and good tolerability	[9,22,25]
Specific carbohydrate diet (SCD)	Restricts complex carbohydrates and processed foods	Microbiome and inflammatory marker changes reported inconsistently	Symptom improvement in some patients; not superior to Mediterranean diet	[12]
Mediterranean diet	Plant-rich, minimally processed dietary pattern	Supports microbial diversity and anti-inflammatory milieu	Comparable symptom control with better adherence	[30]
CD-TREAT	Whole-food mimic of EEN	Similar metabolic and microbial patterns to enteral nutrition	Promising but limited to small pilot studies	[23]
Low-FODMAP diet	Restricts fermentable carbohydrates	Reduces luminal fermentation	Symptom control only; not anti-inflammatory	[26]

Biological pathways underpinning diet-based therapy in Crohn’s disease

Although mechanistic effects are briefly introduced within individual dietary intervention sections, this section synthesises these pathways across interventions to provide an integrated biological framework.

Diet-based therapies in Crohn’s disease exert their effects through a limited number of interconnected biological pathways that converge on intestinal inflammation [13]. Understanding these shared pathways is essential for explaining why certain dietary strategies demonstrate therapeutic potential while others primarily affect symptoms without modifying inflammatory activity.

Dietary Simplification and Reduction of Luminal Antigen Load

A central feature shared by effective diet-based interventions is dietary simplification, characterised by the exclusion of ultra-processed foods, food additives, and complex dietary components that may increase luminal antigenic burden [17]. EEN provides the clearest example of this principle, achieving rapid suppression of intestinal inflammation through complete removal of whole foods [41,42]. Reducing exposure to dietary emulsifiers, maltodextrins, and excess saturated fat may limit disruption of the mucus layer and epithelial surface, thereby decreasing microbial translocation and immune activation [43]. This framework helps explain why structured exclusion-based diets appear more effective than empiric or symptom-driven dietary modifications.

Microbiome Modulation as a Mediator Rather Than a Direct Target 

Current evidence suggests that microbiome modulation functions as a mediator of therapeutic response rather than a direct target [44]. Both EEN and whole-food exclusion diets induce shifts in microbial composition and metabolic activity, often accompanied by reductions in pro-inflammatory taxa and changes in short-chain fatty acid production [44]. These alterations are associated with improvements in inflammatory biomarkers but are heterogeneous across individuals [44].

Importantly, microbial diversity does not consistently increase during successful induction of remission, indicating that restoration of a putatively “healthy” microbiome is not a prerequisite for clinical improvement [23]. Instead, transient suppression of microbial antigenic stimulation during periods of active inflammation may be sufficient to permit mucosal recovery [23]. This perspective cautions against oversimplified interpretations of microbiome data and supports dietary strategies that prioritise functional outcomes over attempts to normalise microbial composition.

Intestinal Barrier Integrity as a Convergent Endpoint 

Improvement in intestinal barrier function represents a key convergent endpoint of effective dietary therapy. Given that intestinal permeability is a recognised feature of Crohn’s disease, limiting exposure to dietary components that impair tight junction function or mucus layer stability may facilitate restoration of epithelial homeostasis [45,46]. 

Implication for Therapeutic Design and Clinical Positioning 

Altogether, these pathways suggest that the efficacy of diet-based therapy depends more on the coordinated modulation of the intestinal environment. Interventions that combine dietary simplification, controlled reintroduction of whole foods, and support for long-term adherence are more likely to achieve durable clinical benefit. This framework explains why structured approaches such as CDED demonstrate greater therapeutic potential than unstructured or highly restrictive diets that are difficult to sustain.

Importantly, these biological pathways support the positioning of diet as an adjunctive therapeutic strategy rather than a replacement for pharmacological treatment, particularly in patients with mild-to-moderate disease. By integrating mechanistic plausibility with clinical feasibility, diet-based therapy can be incorporated into personalised management strategies that align biological effect with patient adherence and quality of life.

Controversies, limitations, and evidence gaps

Despite increasing interest in diet-based therapy for Crohn’s disease, several unresolved challenges continue to limit its consistent integration into routine clinical practice. These challenges do not negate the therapeutic potential of dietary interventions but instead reflect heterogeneity in patient response, methodological limitations in existing studies, and uncertainties surrounding optimal implementation. 

Heterogeneity of Response and Patient Satisfaction 

One of the central challenges in diet-based therapy is the marked variability in clinical response across individuals. While some patients achieve sustained remission with structured dietary interventions, others demonstrate partial response or no benefit [14,26]. Factors contributing to this heterogeneity remain poorly defined but likely include disease phenotype, inflammatory burden, prior treatment exposure, microbiome composition, and baseline dietary patterns [14]. The absence of validated predictors of response limits the ability to individualise dietary therapy and contributes to inconsistent outcomes across studies.

Importantly, most trials have focused on patients with mild-to-moderate disease, restricting generalisability to those with severe, stricturing, or penetrating phenotypes [24]. Until clearer criteria for patient selection are established, dietary interventions should be considered selectively rather than universally applicable.

Adherence, Sustainability, and Real-World Implementation 

Adherence remains a critical determinant of dietary efficacy and a major barrier to real-world implementation. Highly restrictive diets may demonstrate short-term benefits under trial conditions but prove difficult to sustain outside structured research settings [18].

Variability in adherence reporting across studies further complicates the interpretation of outcomes. Without standardised measures of dietary compliance, it is difficult to distinguish true therapeutic failure from inadequate adherence. This challenge underscores the need for pragmatic trial designs that better reflect real-world clinical practice.

Limitations of Current Evidence and Trial Design 

The existing evidence base for diet-based therapy is characterised by relatively small sample sizes, short follow-up durations, and limited use of objective inflammatory endpoints. While reductions in biomarkers such as faecal calprotectin are encouraging, endoscopic outcomes and long-term disease modification remain underexplored. Additionally, much of the strongest evidence derives from paediatric populations, with fewer robust trials conducted exclusively in adults. Heterogeneity in dietary protocols, outcome measures, and comparator arms further limits cross-study comparison. 

Positioning of Diet as Adjunctive Rather Than Replacement Therapy 

A persistent controversy relates to whether diet-based interventions should be viewed as alternatives to pharmacological therapy or as adjunctive strategies. Current evidence supports the latter [17,30,42]. While dietary interventions can induce remission in selected patients, particularly early in the disease course, they are unlikely to replace immunomodulatory or biologic therapy in patients with aggressive or refractory disease [17]. Clear clinical positioning is therefore essential to avoid unrealistic expectations and inappropriate use. Diet-based therapy should be integrated within a multidisciplinary framework, complementing medical treatment rather than competing with it. 

Future research and clinical integration

Advancing diet-based therapy in Crohn’s disease requires a shift from proof-of-concept studies toward research and implementation strategies that address durability, patient selection, and integration within established treatment pathways. While current evidence supports the therapeutic potential of structured whole-food dietary interventions, several priorities must be addressed before such approaches can be more consistently incorporated into routine care.

Strengthening Trial Design and Outcome Measures

Future studies should prioritise longer follow-up periods and the inclusion of objective inflammatory endpoints to better assess the durability of response. Many existing trials focus on short-term induction outcomes, limiting insight into long-term disease control, relapse rates, and mucosal healing. Greater use of endoscopic outcomes, alongside validated biomarkers such as faecal calprotectin, would improve comparability across studies and strengthen conclusions regarding disease modification.

Additionally, further trials conducted exclusively in adult populations are needed, as much of the highest-quality evidence currently derives from paediatric cohorts. Harmonisation of dietary protocols and outcome reporting would also facilitate synthesis across studies and support clearer clinical guidance.

Identifying Predictors of Response to Dietary Therapy

Marked heterogeneity in response highlights the need to identify factors that predict which patients are most likely to benefit from diet-based interventions. Potential predictors may include disease phenotype, inflammatory burden, baseline dietary patterns, or microbial and metabolic features; however, current evidence remains insufficient to guide personalised dietary prescribing. Clarifying predictors of response would allow dietary therapy to be targeted more effectively and reduce unnecessary burden for patients unlikely to benefit.

Optimising Implementation in Multidisciplinary Support

Successful dietary therapy depends not only on biological efficacy but also on feasibility and patient support. Structured whole-food interventions typically require dietitian involvement, patient education, and ongoing monitoring to ensure nutritional adequacy and adherence. Future studies should therefore incorporate implementation outcomes, including acceptability, adherence, and resource requirements, alongside clinical efficacy. Developing scalable models of dietary support will be essential for translating trial findings into real-world practice, particularly in healthcare systems with limited access to specialist nutrition services.

Clinical positioning and practical use

Dietary therapy should be positioned as a structured adjunct within contemporary Crohn’s disease management rather than a replacement for pharmacological treatment. For selected patients with mild-to-moderate disease, structured interventions such as CDED may be considered for induction of remission and, where feasible, continued as part of maintenance strategies with multidisciplinary support. Broader whole-food dietary patterns, including the Mediterranean diet, may be most appropriate as maintenance approaches and as supportive measures alongside medical therapy. In contrast, symptom-directed strategies such as a low-FODMAP diet may improve bloating, pain, and diarrhoea in patients with overlapping functional symptoms, but should not be presented as anti-inflammatory therapy. 

## Conclusions

The evidence reviewed in this article demonstrates that whole-food dietary interventions can influence disease activity through coordinated effects on luminal exposures, microbial activity, and intestinal barrier integrity. However, the therapeutic potential of diet does not lie in universal applicability or replacement of pharmacological treatment, but in targeted integration within existing care pathways. When implemented with appropriate clinical oversight and dietetic support, diet-based interventions offer a biologically plausible and clinically meaningful option for patients with mild-to-moderate disease. 
